# Customized dimetric dimension method for optimized attribute reduction with applications in uncertain clinical information systems^,^

**DOI:** 10.1016/j.mex.2026.104096

**Published:** 2026-08-14

**Authors:** K. Anitha, R. Arunadevi, E. Suganya, S. Sountharrajan

**Affiliations:** a Department of Mathematics, Amrita School of Engineering, Amrita Vishwa Vidyapeetham, Chennai, India; b Department of Mathematics, SRM Institute of Science and Technology, Ramapuram, Chennai, India; c Department of Information Technology, SSN School of Engineering, Shiv Nadar University, Chennai, India; d Department of Computer Science and Engineering, Amrita School of Computing, Amrita Vishwa Vidyapeetham, Chennai, India

**Keywords:** Dimetric dimension, Rough graphs, Metric dimension, Attribute reduction, Reducts, Indiscernibility relation, Structural connectivity

## Abstract

One of the basic problems in dimensionality reduction is determining minimal attribute subsets (reducts) for relational information systems that are not adequately described by traditional tabular methods because of their structural dependencies. This study introduces a new framework of optimized attribute reduction for rough graphs based on rough set theory and metric dimensions of graphs. The proposed methodology translates the information system into a rough graph and proposes four complementary dimetric formulations, namely, Standard Dimetric Distance, Product Dimetric Distance, Normalized Rough Degree Metric and Rough Membership-Weighted Dimetric, to describe the structural connectivity as well as the uncertainty. The basis with the minimum size of representative vertices that fully characterize vertices by their dimetric representations is then calculated, and this basis is called dimetric basis.The minimum set of representative vertices that fully characterise all vertices by their dimetric representations is then computed, which is called dimetric basis, and is used for efficient generation of relational reducts. The mathematical properties of the proposed formulations are proved for basic rough graph families, such as paths, cycles and complete graphs. The clinical applicability of the framework is illustrated through two clinical benchmark datasets, where a data reduction of up to 80 percent can be achieved with the proposed dimetric reducts without deteriorating classification performance and even with an improvement in it. The proposed framework is interpretable and has computation efficiency and is a flexible basis for uncertainty-aware graph analysis in complex information systems through the combination of structural graph information and rough approximations.

## Graphical abstract

**Figure:**
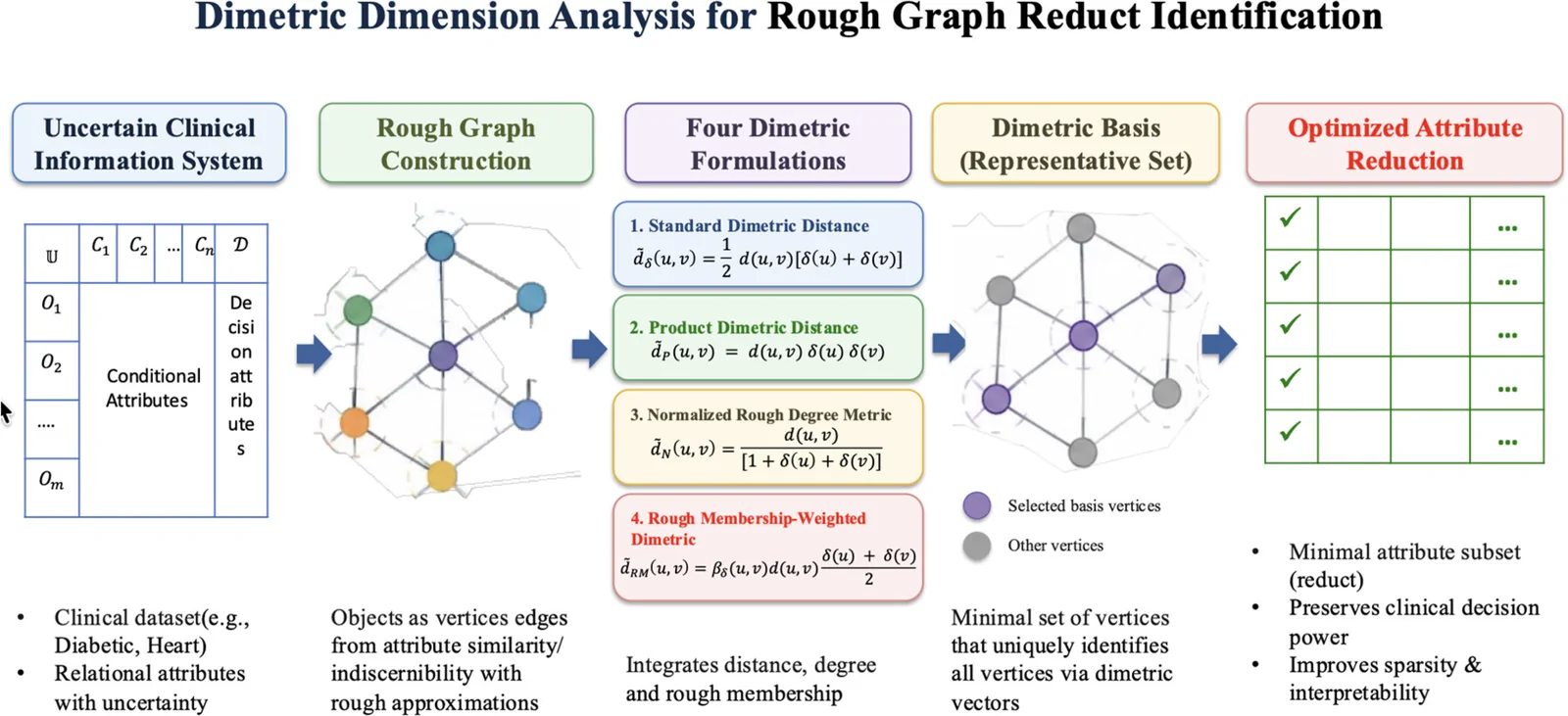

**Specifications table**

**Table:** 

**Subject area**	Mathematics and Statistics
**More specific subject area**	Discrete Mathematics, Graph Theory, and Rough Set Theory
**Name of your method**	Dimetric Dimension Analysis for Rough Graph Reducts
**Name and reference of original method**	Metric Dimension of Graphs and Rough Set Theory
**Resource availability**	UCI Machine Learning Repository (Heart Disease datasets)

## Background

Since long ago, uncertainty and vagueness in complex information systems has been a topic of discussion in the field of data science and computational intelligence. In the early 1980s the theory of rough sets was developed by Zdzisław Pawlak [1] to provide a powerful mathematical tool to deal with indiscernibility by mean of extending a set into two approximations: the lower and the upper sets, which are constructed by equivalence relations. This theory mostly works well in attribute reduction, whereby the aim is to determine the minimal subsets of characteristics or reducts, which have the same partitioning ability as the original full set of attributes [2].

Although classical rough set tools are efficient in tabular data, most recent datasets are relational in nature and can be described as a graph. In such systems, the objects are not just rows in a table but nodes in a network with the edges depicting the common attributes or relationships. This has resulted in rough graphs, an extension of standard graph theory that identifies approximations with sets of vertices or edges based on relation of indiscernibility [3,4]. The more recent developments have incorporated the use of fuzzy rough, intuitionistic and neutrosophic rough graphs in managing multi-valued uncertainty in social, biological, and transportation networks [[5], [6], [7]].

A significant gap remains, however, in how structural distinguishability is quantified within these rough structures. In classical graphs, metric dimension theory has been extensively studied in the context of determining a smallest subset of vertices, called the metric basis, such that the distance vectors of all vertices in the graph can be uniquely determined from those of the basis vertices [8]. But the common concept of dimension of a metric doesn't take into account the rough approximations or the varying importance of nodes according to their degree of connectivity. For instance, in a real-world application such as clinical decision support, or network anomaly detection, the significance of a node can depend not only on how close it is to a "hub," but also on the strength of its local connectivity [9,10]. The motivating idea behind the dimetric methodology is to balance these conflicting views. The dimetric dimension is a new visualization and characterization of uncertain networks through a rough set paradigm, combining vertex degree and shortest-path distance. This can be used to find reducts that are minimal both mathematically and structurally, and the decisionmakers can be sure they are selecting the most influential and discriminative objects in the system. Slater [11] and Harary and Melter [12] independently proposed the idea of metric dimension for recognizing vertices of graphs by distance representations. Later, several theoretical works have further generalized the concept of metric dimension for various other families of graphs and applications such as network optimization, structural graph analysis and combinatorial problems [12,13]. But, these works are mainly focused on crisp graph structures represented by shortest path distances and do not directly consider uncertainty in the form of rough graphs. This restriction encourages the research of the proposed dimetric model of rough-based attribute reduction for rough graphs.

### Scientific novelty and contribution

While rough graph theory and metric dimension studies have been separately investigated, the combination of them for attribute reduction of structural information in the uncertain environment is not extensively studied. The existing metric dimension techniques are mostly based on the shortest-path distances, while classical rough set-based attribute reduction is related to indiscernibility relations and particularly does not use the inherent structure of graph representations. In order to fill this gap, this work suggests a customized dimetric framework for rough graphs which considers both the graph topology and uncertainty information. The principal mathematical contributions of this work are summarized as follows:

• Four complementary formulations of the Standard Dimetric Distance, namely Standard Dimetric Distance, Product Dimetric Distance, Normalized RoughDegree Metric and Rough Membership-Weighted Dimetric, are proposed for rough graphs, to represent different structural characteristics of uncertain graph-based information systems.
• The proposed dimetric formulations combine the shortest-path distance, the degree of vertices and rough membership information in a single framework for defining a structural distinguishability metric in the rough graph environment, which extends the classical notion of the metric dimension.
• Theoretical characterization of the proposed framework for fundamental rough graph families (rough paths, rough cycles and rough complete graphs) is set up, providing a mathematical basis for the proposed methodology.
• In view of the above, an attribute reduction algorithm based on graph is proposed to select structurally significant discriminator sets, so that the optimized reducts can be generated.
• The proposed methodology is validated on illustrative and real-world clinical datasets to prove its effectiveness in preserving the discriminative capability whilst reducing redundancy within the proposed structural reducts.
• In the present study, the proposed new structural perspective on attribute reduction is introduced, which can be used in uncertain graph-based information systems other than the clinical datasets used in this study.
• In this work, a novel dimetric framework is proposed, which contains four complementary formulations: Standard Dimetric Distance, Product Dimetric Distance, Normalized Rough Degree Metric and Rough Membership-Weighted Dimetric, for rough graph structural distinguishability modelling.

The mathematical basis for the subsequent theoretical analysis are the proposed dimetric formulations, in which the dimension of the fundamental rough graph classes is determined, and then used to optimized attribute reduction.

## Method details

### Mathematical foundations of rough sets and graphs

To implement the dimetric dimension, one must first establish the foundational properties of the information system and its graph transformation. The process begins with a universe of objects U, along with an indiscernibility relation B⊆U, which signifies our limited understanding of the elements in U. Let X denote a subset of U. The set X in relation to B is defined as follows:

• Lower Approximation: $B_{*}\left(X\right)=\left\{\overset{‾}{x}\in U:B\left(\overset{‾}{x}\right)\subseteq X\right\}$
• Upper Approximation: $B^{*}\left(X\right)=\left\{\overset{‾}{x}\in U:B\left(\overset{‾}{x}\right)\cap X\neq \emptyset \right\}$

### Indiscernibility and approximations

The process of lowering an information system to the point where its collection of attributes becomes independent and it is no longer possible to delete any more characteristics without losing some information from the system is known as identifying a reduct. Another way to describe rough sets is to use a rough membership function, which is specifically stated as follows:

$$\mu_{X}^{B}\left(\overset{‾}{x}\right)=\frac{\left|{\left[\overset{‾}{x}\right]}_{B}\cap X\right|}{\left|{\left[\overset{‾}{x}\right]}_{B}\right|},\;\text{where}\;\mu_{X}^{B}\left(\overset{‾}{x}\right)\in \left[0,1\right]$$

Where $\mu_{X}^{B}\left(\overset{‾}{x}\right)\;$captures the degree to which an object belongs to a set given its indiscernibility class.

**Rough Graph Modeling** [14]

A data table can be transformed into a rough graph by designating the attribute set P and the corresponding set of objects U, thereby forming an appropriate set of vertices. $\overset{˜}{v}=\left\{{\overset{˜}{v}}_{1},{\overset{˜}{v}}_{2},\ldots ,{\overset{˜}{v}}_{\left|U\right|}\right\}$ is linked to the set of edges $E=\cup e_{\overset{˜}{k}}\left({\overset{˜}{v}}_{i},{\overset{˜}{v}}_{j}\right)$. Different equivalence classes ${\left[\overset{˜}{e}\right]}_{B}$ can be constructed from this E. If Y is the sum of certain ${\left[\overset{˜}{e}\right]}_{B}$, then any subgraph T=(W,Y), where W⊆V,Y⊆E, is referred to as a B-definable graph or B-exact graph. In contrast, a graph T is referred to as a B-undefinable graph or B-rough graph when it is not possible to describe it accurately in this way. Two approximate graphs, $B{\left(T\right)}_{*}=(W,B{\left(Y\right)}_{*}$) and $B{\left(T\right)}^{*}=\left(W,B{\left(Y\right)}^{*}\right)$, can be used in these situations for an approximation as follows: $B{\left(Y\right)}_{*}=\left\{\overset{˜}{e}\in E:{\left[\overset{˜}{e}\right]}_{B}\subseteq Y\right\},\;B{\left(Y\right)}^{*}=\left\{\overset{˜}{e}\in E:{\left[\overset{˜}{e}\right]}_{B}\cap Y\neq \emptyset \right\}$ We refer to the graphs $B{\left(T\right)}_{*}$ and $B{\left(T\right)}^{*}$ as the B-lower and B-upper approximation graphs of T, respectively. The graph $\left(B{\left(T\right)}_{*},B{\left(T\right)}^{*}\right)$ is referred to as a B-rough graph. The edge set Y of 's B-boundary is the set $bn_{B(Y)}=B{\left(Y\right)}_{*}-B{\left(Y\right)}^{*}$.

**Rough weighted Graph** [15] A mapping that assigns a real number to each edge in the universe graph U=(V,E) is represented as $\overset{˜}{v}=\left\{{\overset{˜}{v}}_{1},{\overset{˜}{v}}_{2},\ldots ,{\overset{˜}{v}}_{\left|V\right|}\right\}$, $E=\;\overset{\left|E\right|}{\underset{k=1}{\cup }}{\overset{˜}{e}}_{k}\left({\overset{˜}{v}}_{i},{\overset{˜}{v}}_{j}\right)$. The edge weight of $\overset{˜}{e},\omega \left({\left[{\overset{˜}{e}}_{uv}\right]}_{B}\right)=f\left(\omega (\overset{˜}{e})\right),A$ is defined as $\forall \overset{˜}{e}\in E,\omega :\overset{˜}{e}\to \omega \left(\overset{˜}{e}\right)$, the real number $\omega (\overset{˜}{e})$. The class weight refers to the edge equivalence class ${\left[{\overset{˜}{e}}_{uv}\right]}_{B}$, when $\overset{˜}{e}\in {\left[{\overset{˜}{e}}_{uv}\right]}_{B}$. The set of graphs with the class weight corresponding to their edge equivalence classes is called a rough weighted graph.

**Rough Graph** [16] Consider the triple R={V,E,ω}, which is made up of non-empty sets. $V=\{{\overset{˜}{v}}_{1},{\overset{˜}{v}}_{2},\ldots ,{\overset{˜}{v}}_{n}\}=U$, where U is a universe, ω is a function ω:V→[0,1] and $E=\{{\overset{˜}{e}}_{1},{\overset{˜}{e}}_{2},\ldots ,{\overset{˜}{e}}_{m}\}$ is an edge set for V.

$$R\left({\overset{˜}{v}}_{i},{\overset{˜}{v}}_{j}\right)=\left\{ \begin{array}{cc} \;\text{If}\;\text{max}\left(\omega_{G}^{V}\left({\overset{˜}{v}}_{i}\right),\omega_{G}^{V}\left({\overset{˜}{v}}_{j}\right)\right)>0, & \;\text{then}\;{\overset{˜}{v}}_{i}{\overset{˜}{v}}_{j}\;\text{exists}\;\\ \;\text{If}\;\text{max}\left(\omega_{G}^{V}\left({\overset{˜}{v}}_{i}\right),\omega_{G}^{V}\left({\overset{˜}{v}}_{j}\right)\right)=0, & \;\text{no}\;\text{edge}\; \end{array}\right.$$

Existing metric dimension approaches distinguish vertices using shortest-path distance representations [[11], [12], [13],^17^]. In uncertain graph environments, however, shortest-path distance alone may not adequately capture structural characteristics associated with rough graph representations. Therefore, the proposed dimetric framework introduces four complementary formulations that integrate topological distance, vertex connectivity, and rough membership information.

### Discretization of continuous attributes

Prior to constructing the rough graph, continuous clinical attributes were discretized to establish the indiscernibility relations required by rough set theory. A consistent discretization strategy was employed throughout all experiments to ensure uniform construction of equivalence classes and fair comparison of the proposed dimetric formulations. The resulting indiscernibility relations form the basis for rough graph construction and the subsequent computation of the proposed dimetric measures.

### Proposed dimetric metric framework

The dimetric framework proposed in this work extends the classical shortest-path distance by incorporating additional structural characteristics of rough graphs for attribute reduction. Unlike conventional metric distance, which relies solely on graph distances, the proposed formulations integrate shortest-path distance with vertex degree and rough membership information to capture structural distinguishability under uncertainty. The term "dimetric" is introduced in this work to denote this integrated distance framework. Four complementary formulations are proposed to accommodate different structural characteristics encountered in rough graph-based information systems.

### Formulation 1: standard dimetric distance

The Standard Dimetric Distance is introduced in this work as the baseline formulation of the proposed dimetric framework. It combines the shortest-path distance and the average degree of adjacent vertices to quantify structural distinguishability in rough graphs. The term "standard" refers only to the reference or baseline formulation proposed in this study and does not imply the existence of an established standard definition in the existing literature. The remaining formulations are extensions of this baseline measure developed for different graph characteristics.

The standard dimetric distance between two vertices $\overset{˜}{a}$ and $\overset{˜}{b}$ is the product of their shortest path length and the average of their degrees:

$${\overset{˜}{d}}_{\delta }\left(\overset{˜}{a},\overset{˜}{b}\right)=\frac{1}{2}\cdot d\left(\overset{˜}{a},\overset{˜}{b}\right)\left(\delta \left(\overset{˜}{a}\right)+\delta \left(\overset{˜}{b}\right)\right)$$

*Where* δ(v) is the vertex degree and $\overset{˜}{d}\left(\overset{˜}{a},\overset{˜}{b}\right)$ is the shortest path edge count. The Standard Dimetric Distance serves as the baseline structural measure for identifying distinguishable vertices in rough graphs. It provides the foundation upon which the remaining dimetric formulations are developed. The integration of shortest-path distance and vertex degree enables vertices with both high connectivity and favourable structural positioning to be distinguished more effectively than when shortest-path distance alone is considered.

While the Standard Dimetric Distance provides a general structural measure, different application scenarios may require greater emphasis on connectivity or uncertainty. Therefore, three additional dimetric formulations are proposed as extensions of the baseline formulation.

**Formulation 2: Product Dimetric Distance**: In the systems where connectivity is the most relevant factor, product-based measure is used:

$$PDD={\overset{˜}{d}}_{PDD}\left(\overset{˜}{a},\overset{˜}{b}\right)=\delta \left(\overset{˜}{a}\right)\delta \left(\overset{˜}{b}\right)\overset{˜}{d}\left(\overset{˜}{a},\overset{˜}{b}\right).$$

It works quite well especially in communication networks where highly degree nodes are isolated critical relay points.

In weighted rough graphs, uncertainty associated with boundary regions influences structural connectivity. The following normalized formulation incorporates this information into the dimetric measure.

**Formulation 3: Normalized Rough Degree Metric** The degree is adjusted in a similar way in weighted rough graphs:

$$\frac{1}{2}\left(d\left(\overset{˜}{a},\overset{˜}{b}\right)\left(\delta \left(\overset{˜}{a}\right)+\delta \left(\overset{˜}{b}\right)\right)\right)={\overset{˜}{d}}_{\delta }\left(\overset{˜}{a},\overset{˜}{b}\right)={\overset{˜}{d}}_{NR}\left(\overset{˜}{a},\overset{˜}{b}\right).$$

This is a combination of both the rough set and the boundary information into the topological distance, which is the distance d(u,v)between two vertices u and v defined as length of the shortest path connecting them, measured as the minimum number of edges traversed between the two vertices. If no path exists, the distance is considered undefined (or infinite in disconnected graphs). In the proposed framework, this shortest-path distance forms the topological component of the dimetric formulations.

Since not all relationships possess the same degree of certainty, the final formulation integrates rough membership values to weight structural contributions according to the confidence of the corresponding rough graph representation**.**

**Formulation 4: Rough Membership-Weighted Dimetric** In order to introduce semantic certainty the degree influence is weighted by the edge rough membership $\omega \left(\overset{˜}{a},\overset{˜}{b}\right)=\text{min}\left(\omega \left(\overset{˜}{a}\right),\omega \left(\overset{˜}{b}\right)\right)$

$$\overset{˜}{{\overset{˜}{d}}_{RM}\left(\overset{˜}{a},\overset{˜}{b}\right)=\;d}\left(\overset{˜}{a},\overset{˜}{b}\right)+\omega \left(\overset{˜}{a},\overset{˜}{b}\right)\left(\delta \left(\overset{˜}{a}\right)+\delta \left(\overset{˜}{b}\right)\right)$$

This ensures that vertices with high membership certainty contribute more to the distinguishability of the basis.This makes sure that vertices that have a high membership certainty have a greater contribution to the distinguishability of the basis.

Hence

• Formulation 1 describes Baseline structural measure.
• Formulation 2 deals with Connectivity-focused extension.
• Formulation 3 incorporates uncertainty through rough graph normalization.
• Formulation 4 adds semantic confidence via rough membership weighting.

**Remark:** The four proposed dimetric formulations are designed for different structural characteristics of rough graphs. The Standard Dimetric Distance serves as the baseline formulation, whereas the remaining three formulations extend the framework by emphasizing connectivity, normalized structural information, and rough membership uncertainty, respectively.

### Theoretical characterization of graph topologies

#### Dimetric vector

Let the triple R={V,E,ω}, which is made up of non-empty sets be the Rough graph with $V=\{{\overset{˜}{v}}_{1},{\overset{˜}{v}}_{2},\ldots ,{\overset{˜}{v}}_{n}\}=U$, where U is a universe, ω is a function ω:V→[0,1] and $E=\{{\overset{˜}{e}}_{1},{\overset{˜}{e}}_{2},\ldots ,{\overset{˜}{e}}_{m}\}$ is an edge set for V. Let $W=\{{\overset{˜}{w}}_{1},{\overset{˜}{w}}_{2},\ldots ,{\overset{˜}{w}}_{k}\}\subseteq V$ be an ordered subset of vertices. The **dimetric vector** of a vertex $\tilde{v\;}\;\in \;V$ with respect to W is defined as

$$r_{\delta }\left(\tilde{v\;}|\;W\right)=\left[\tilde{d}\;\left(\tilde{v\;},{\overset{˜}{w}}_{1}\right),\tilde{d}\;\left(\tilde{v\;},{\overset{˜}{w}}_{2}\right),\ldots \tilde{d}\;\left(\tilde{v\;},{\overset{˜}{w}}_{k}\right)\;\right].$$

$\tilde{d}\;\left(\tilde{v\;},{\overset{˜}{w}}_{i}\right)$ denotes the proposed Standard Dimetric Distance between $\tilde{v\;}$ and ${\overset{˜}{w}}_{i}$. Two vertices are said to be **dimetrically distinguishable** if their corresponding dimetric vectors are different.

Remark: The proposed dimetric representation associates each vertex $\tilde{v}\;\in \;V$ with the signature $\phi \left(\tilde{v}\;\right)=\left(d\left(\tilde{v},.\right)\deg\left(\tilde{v}\;\right),\;\mu^{B}(\tilde{v}\right))$ where $d(\tilde{v},.)$ denotes the proposed dimetric distance vector, $\deg(\tilde{v}\;)$ represents the vertex degree and $\mu^{B}\left(\tilde{v}\right)$ is the corresponding rough membership value.

The mapping ϕ is injective whenever every pair of distinct vertices differs in at least one of these three components. In other words, for any distinct vertices $\tilde{u},\tilde{v}\;\in \;V,$$\deg\left(\tilde{u},.\;\right)\neq \deg\left(\tilde{v},.\;\right)$ or $\deg\left(\tilde{u}\;\right)\neq \deg\left(\tilde{v}\;\right)$ or $\mu^{B}\left(\tilde{u}\right)\neq \mu^{B}\left(\tilde{v}\right)$ Under these conditions, every vertex possesses a unique dimetric signature, enabling the construction of a unique dimetric resolving set. In highly symmetric graph structures, however, distinct vertices may possess identical distance vectors, identical degrees, and identical rough membership values. Such vertices produce identical dimetric signatures and cannot be distinguished solely by the proposed representation. Although these pathological cases rarely arise in practical clinical information systems due to heterogeneous patient characteristics and varying rough memberships, extending the framework to explicitly distinguish structurally equivalent vertices remains an interesting direction for future research.

### Dimetric resolving set

A subset W⊆V is called a dimetric resolving set of the rough graph R if every pair of distinct vertices possesses distinct dimetric vectors with respect to W

### Dimetric dimension

Let the triple R={V,E,ω}, which is made up of non-empty sets be the Rough graph with $V=\{{\overset{˜}{v}}_{1},{\overset{˜}{v}}_{2},\ldots ,{\overset{˜}{v}}_{n}\}=U$, where U is a universe, ω is a function ω:V→[0,1] and $E=\{{\overset{˜}{e}}_{1},{\overset{˜}{e}}_{2},\ldots ,{\overset{˜}{e}}_{m}\}$ is an edge set for V. Let W⊆V be a dimetric resolving set of R*.* The dimetric dimension of the rough graph R*,* denoted by $\beta_{\delta }\left(R\right)$ is defined as the minimum cardinality of all dimetric resolving sets of R. That is,

$$\beta_{\delta }\left(R\right)=min\left\{\left|W\right|\;:\;W\;is\;a\;dimetric\;resolving\;set\;of\;R\right\}.$$

The following theorems establish the dimetric dimension of fundamental rough graph classes under the proposed dimetric framework. Unlike the classical metric dimension, which relies solely on shortest-path distances, the present analysis is based on the proposed dimetric formulations that integrate topological distance with structural characteristics of rough graphs. To the best of our knowledge, these theoretical characterizations have not been reported in the existing literature and constitute the mathematical foundation of the proposed graph-based attribute reduction methodology.

Classical metric dimension theory establishes resolving sets using shortest-path distance representations in crisp graphs [[11], [12], [13],^17^]. The following theorems extend this concept by establishing the dimetric dimension of rough graph classes under the proposed dimetric framework, thereby providing the theoretical basis for structural attribute reduction in uncertain information systems.

Theorem 1 *Let* $R(P_{\overset{˜}{n}})$ *be a path rough graph. Then* $\beta_{\delta }\left(R(P_{\overset{˜}{n}})\;\right)=1$

Proof. If $R(P_{\overset{˜}{n}})\;$be a path rough graph, the degree of each vertex will be $\delta \left({\overset{˜}{n}}_{1}\right)=\delta \left({\overset{˜}{n}}_{t}\right)$ and $\delta \left({\overset{˜}{n}}_{2}\right)=\cdots =\delta \left({\overset{˜}{n}}_{n-1}=1\right)$. The distance measured in degrees is described as ${\overset{˜}{d}}_{\delta }\left({\overset{˜}{v}}_{1},{\overset{˜}{v}}_{2}\right)=\frac{1}{2}\left(d\left({\overset{˜}{v}}_{1},{\overset{˜}{v}}_{2}\right)\left(\delta \left({\overset{˜}{v}}_{1}\right)+\delta \left({\overset{˜}{v}}_{2}\right)\right)\right)$

The dimetric vector depicting R with respect to A is divided into three distinct instances:

Case(i): If $A=\{{\overset{˜}{n}}_{1}\}$, then $\beta_{\delta }\left(R(P_{\overset{˜}{n}})\;\right)=1$, for $\overset{˜}{n}=6,8,10,\ldots 2n$ and $\overset{˜}{n}=3$. The dimetric representation of vector R w.r.t A

$$r\left({\overset{˜}{v}}_{\alpha }|A\right)=\left\{ \begin{array}{cc} 0 & ,\;\text{when}\;\alpha =1\\ 1.5 & ,\;\text{when}\;\alpha =2\\ 3 & ,\;\text{when}\;\alpha =3\\ n+m & ,\;\text{when}\;\alpha =n-1\\ n-1 & ,\;\text{when}\;\alpha =n \end{array}\right.$$

${\overset{˜}{v}}_{1}$ will be determined by set $P_{\overset{˜}{n}}(\overset{˜}{n}=6,8,\ldots ,2n$ and $\overset{˜}{n}=3)$.

**Case (ii):** If $A=\{{\overset{˜}{v}}_{2}\}$, then $\beta_{\delta }\left(R(P_{\overset{˜}{n}})\;\right)=1$, for $\overset{˜}{n}=5,7,\ldots ,2n+1$. The dimetric representation vector of $P_{\overset{˜}{n}}$ w.r.t A will be

$$r\left({\overset{˜}{v}}_{\alpha }|A\right)=\left\{ \begin{array}{cc} 1.5 & ,\;\text{when}\;\alpha =1\\ 0 & ,\;\text{when}\;\alpha =2\\ 2 & ,\;\text{when}\;\alpha =3\\ \ldots & ,\;\text{when}\;\alpha =n-1\;\text{where}\;m=0,1,2\\ 2m & ,\;\text{when}\;\alpha =n \end{array}\right.$$

The discriminator set $R\left(P_{\overset{˜}{n}}\right)\left(\overset{˜}{n}=5,7,\ldots ,2n+1\right)$ will be ${\overset{˜}{v}}_{2}$.

**Case (iii):** If $A=\{{\overset{˜}{v}}_{3}\}$, then $\beta_{\delta }\left(R(P_{\overset{˜}{n}})\;\right)=1$, for $\overset{˜}{n}=4$. The dimetric vector representation of $P_{4}$ w.r.t A will be

$$r\left(v_{\alpha }|U\right)=\left\{ \begin{array}{cc} 3 & ,\;\text{when}\;\alpha =1\\ 2 & ,\;\text{when}\;\alpha =2\\ 0 & ,\;\text{when}\;\alpha =3\\ 1.5 & ,\;\text{when}\;\alpha =4 \end{array}\right.$$

The discriminator set $R\left(P_{\overset{˜}{n}}\right)\left(\overset{˜}{n}=4\right)$ will be ${\overset{˜}{v}}_{3}$.

**Fig. 1:**
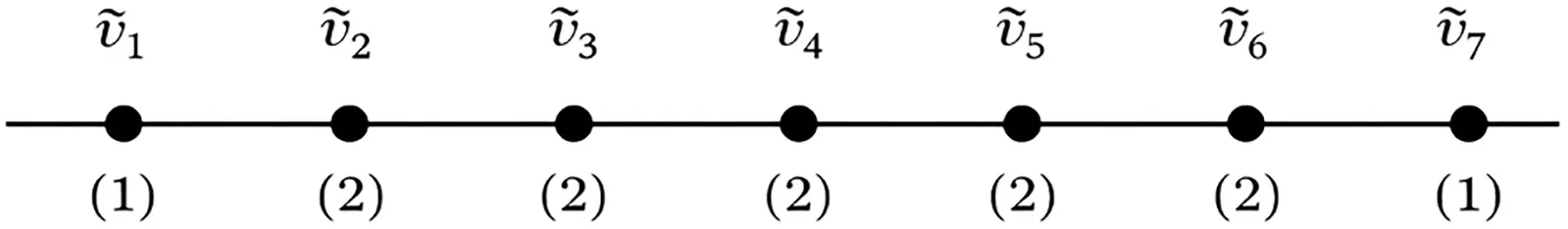
Rough path with 7 vertices $R(P_{7})$.

For example Path graph with 7 vertices $P_{7}$ the edge set will be $\left\{{\overset{˜}{e}}_{1},{\overset{˜}{e}}_{2},\ldots ,{\overset{˜}{e}}_{6}\right\}$, while the vertex set $P_{7}$ will be $\left\{{\overset{˜}{v}}_{1},{\overset{˜}{v}}_{2},\ldots ,{\overset{˜}{v}}_{7}\right\}$. $\left\{{\overset{˜}{v}}_{2}\right\}$ will be the discriminator set $R(P_{7})$. Table 1 provides the representation vector of dimetric for $R(P_{7})$ with respect to A. Fig. 1

**Table 1:** Dimetric vector of $R(P_{7})$ with respect to $A=\{{\overset{˜}{v}}_{2}\}$.

d(.,).	${\overset{˜}{v}}_{1}$	${\overset{˜}{v}}_{2}$	${\overset{˜}{v}}_{3}$	${\overset{˜}{v}}_{4}$	${\overset{˜}{v}}_{5}$	${\overset{˜}{v}}_{6}$	${\overset{˜}{v}}_{7}$
$A=\{{\overset{˜}{v}}_{2}\}$	1.5	0	2	4	6	8	7.5

Remark : Unlike the classical metric dimension results for path graphs reported in [[11], [12], [13],^17^], Theorem 1 establishes the dimetric dimension of rough path graphs under the proposed dimetric framework.

Theorem 2 *Let* $R(C_{\overset{˜}{n}})$ *be cycle rough graph, then* $R(\beta_{\delta }\left(C_{\overset{˜}{n}}\right))=2$.

**Proof.** Each vertex's degree will be two since $R(C_{\overset{˜}{n}})$ is a 2 -regular graph, where ${\overset{˜}{v}}_{1},{\overset{˜}{v}}_{2},\ldots ,{\overset{˜}{v}}_{r}\in R\left(C_{\overset{˜}{n}}\right)$, the dimetric is $\frac{1}{2}\left(d\left({\overset{˜}{v}}_{1},{\overset{˜}{v}}_{2}\right)\left(\delta \left({\overset{˜}{v}}_{1}\right)+\delta \left({\overset{˜}{v}}_{2}\right)\right)\right)$. There are two situations for the dimetric representation vector of $R(C_{\overset{˜}{n}})$ with respect to the discriminator set A. Table 2

**Table 2:** Dimetric vector of $C_{6}$ with respect to $A=\{{\overset{˜}{v}}_{1},{\overset{˜}{v}}_{2}\}$.

d(.,).	${\overset{˜}{v}}_{1}$	${\overset{˜}{v}}_{2}$	${\overset{˜}{v}}_{3}$	${\overset{˜}{v}}_{4}$	${\overset{˜}{v}}_{5}$	${\overset{˜}{v}}_{6}$
$A=\{{\overset{˜}{v}}_{1},{\overset{˜}{v}}_{2}\}$	0,2	2,0	4,2	6,4	4,6	2,4

Case (i): For $R\left(C_{\overset{˜}{n}}\right)\left(\overset{˜}{n}=4,5,\ldots \right)$ the resolving will be $\{{\overset{˜}{v}}_{1},{\overset{˜}{v}}_{2}\}=A$. The degree distance representation is given as

$$r\left({\overset{˜}{v}}_{\alpha }|A\right)=\left\{ \begin{array}{cc} \left(0,2\right) & ,\;\text{when}\;\alpha =1\\ \left(2,0\right) & ,\;\text{when}\;\alpha =2\\ \left(2m,m\right) & ,\;\text{when}\;\alpha =3\\ \ldots .. & ,\;\text{when}\;\alpha =n-1\\ \left(2m,3m\right) & ,\;\text{when}\;\alpha =n\\ \left(2m,2m\right) & ,\ldots \end{array}\right.$$

$$∴R\left(\beta_{\delta }\left(C_{\overset{˜}{n}}\right)\right)=2\;\text{for}\;\overset{˜}{n}=4,5,\ldots$$

Case (ii): For $C_{3}$, the dimetric representation is given as

$$r\left({\overset{˜}{v}}_{\alpha }|A\right)=\left\{ \begin{array}{cc} \left(0,2\right) & ,\;\text{when}\;\alpha =1\\ \left(2,0\right) & ,\;\text{when}\;\alpha =2\\ \left(2,2\right) & ,\;\text{when}\;\alpha =3 \end{array}\right.$$

$$∴R\left(\beta_{\delta }\left(C_{\overset{˜}{n}}\right)\right)=2\;\text{for}\;\overset{˜}{n}=3$$

**Fig. 2:**
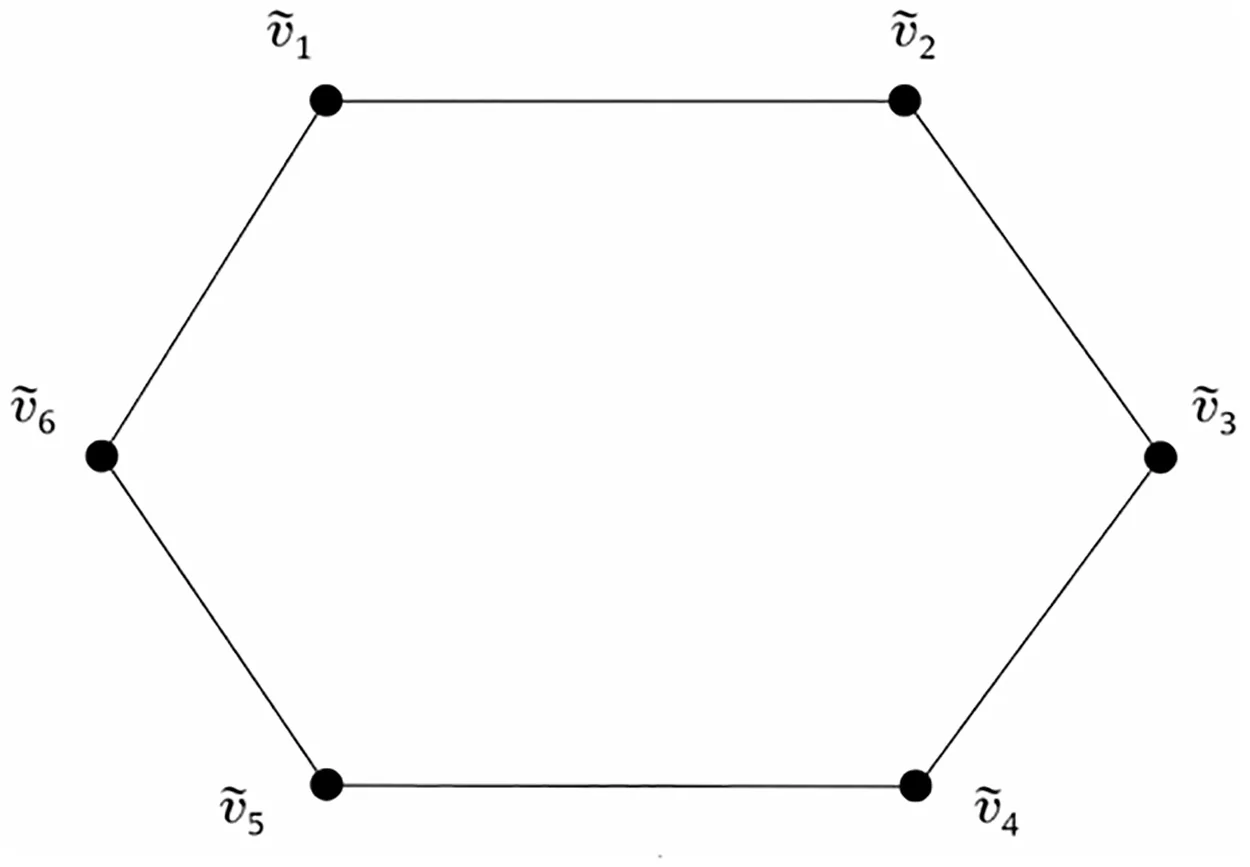
Structure of Rough Cycle $R(C_{6})$.

Example 3. Fig. 2 illustrates $R(C_{6})$ with $V\left(C_{6}\right)=\left\{{\overset{˜}{v}}_{1},{\overset{˜}{v}}_{2},\ldots ,{\overset{˜}{v}}_{6}\right\}$ and $E\left(C_{6}\right)=\left\{{\overset{˜}{e}}_{1},{\overset{˜}{e}}_{2},\ldots ,{\overset{˜}{e}}_{6}\right\}$. Table 3 provides the vector representation of dimetric for $R(C_{6}$) with respect to $A.\{{\overset{˜}{v}}_{1},{\overset{˜}{v}}_{2}\}$ is the discriminator set for $R(C_{6})$.

$$∴R\left(\beta_{\delta }\left(C_{6}\right)\right)=2$$

**Table 3:** Degree-distance vector $K_{6}$ w.r.t A.

d(.,).	$U=\{{\overset{˜}{v}}_{1},{\overset{˜}{v}}_{2},\ldots ,{\overset{˜}{v}}_{5}\}$
${\overset{˜}{v}}_{1}$	(0,5,5,5,5)
${\overset{˜}{v}}_{2}$	(5,0,5,5,5)
${\overset{˜}{v}}_{3}$	(5,5,0,5,5)
${\overset{˜}{v}}_{4}$	(5,5,5,0,5)
${\overset{˜}{v}}_{5}$	(5,5,5,5,0)
${\overset{˜}{v}}_{6}$	(5,5,5,5,5)

This process extends the proposed dimetric framework to rough cycle graphs. This result demonstrates that the proposed dimetric formulation remains applicable to cyclic graph structures while preserving structural distinguishability, thereby extending conventional metric dimension analysis to rough graph environments.

Theorem 3 *Let* $R(K_{\overset{˜}{n}})$ *be a complete rough graph. Then* $R(\beta_{\delta }\left(K_{\overset{˜}{n}}\right))=n-1$.

**Proof.** The degree of $R(K_{\overset{˜}{n}})$ will be (n−1); the length of the shortest path between any two vertices will be 1. The degree distance vertex representation of $K_{\overset{˜}{n}}$ w.r.t A will be

$$r\left({\overset{˜}{v}}_{\alpha }|A\right)=\left\{ \begin{array}{cc} \left(0,n-1,n-1,\ldots ,n\left(n-1\right)\right) & ,\;\text{when}\;\alpha =1\\ \left(n-1,0,n-1,\ldots ,n\left(n-1\right)\right) & ,\;\text{when}\;\alpha =2\\ \left(2m,m\right) & ,\;\text{when}\;\alpha =3\\ \ldots \ldots . & ,\;\text{when}\;\alpha =n\\ \left(\left(n-1\right),\left(n-1\right),\left(n-1\right),\ldots ,\left(n-1\right)\left(n-1\right),0\right) & \end{array}\right.$$

$$∴R\left(\beta_{\delta }\left(K_{\overset{˜}{n}}\right)\right)=n-1.$$

**Fig. 3:**
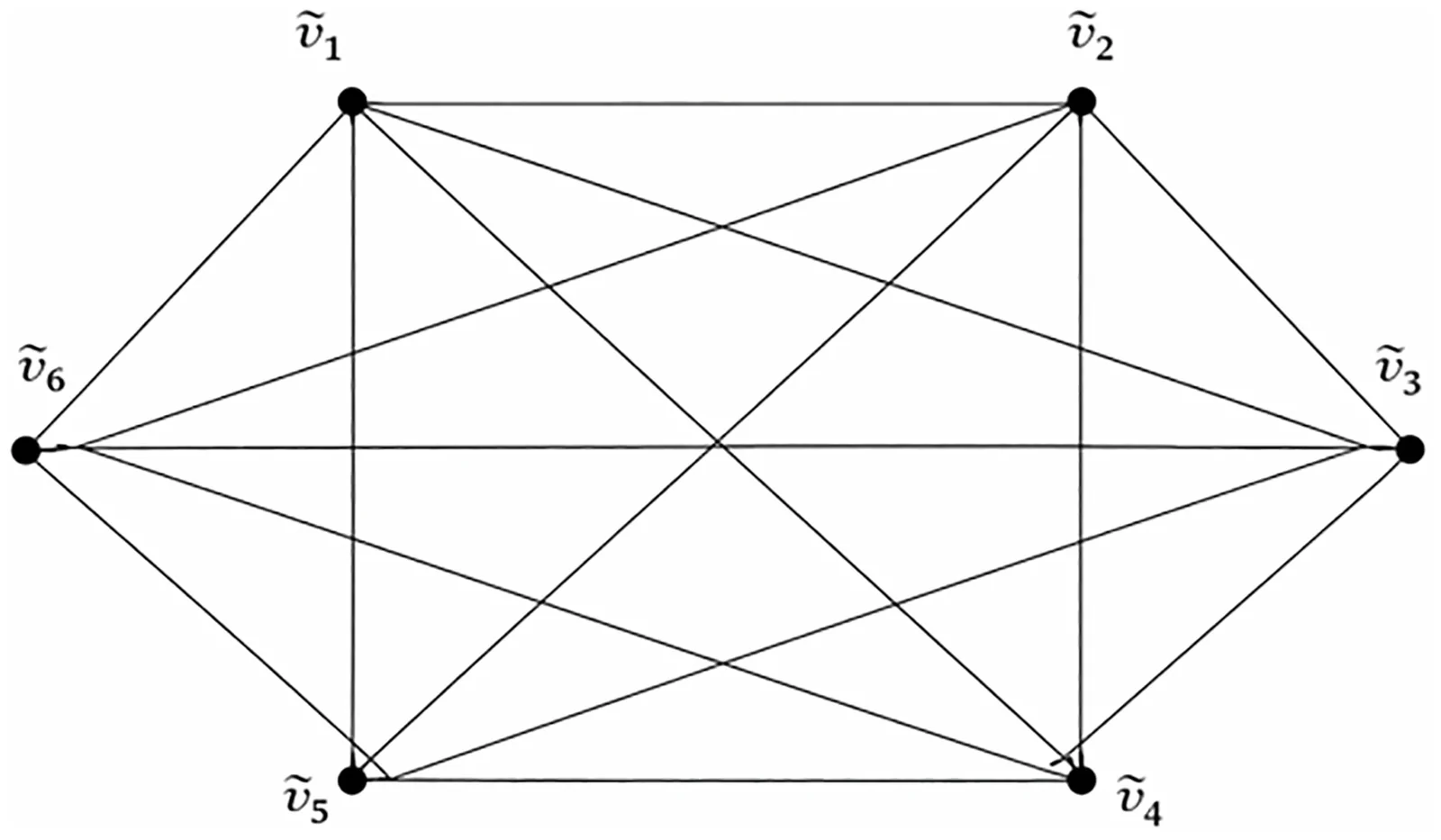
Structure of Rough complete graph.

The complete rough graph depicted in Fig. 3 which has six vertices and fifteen edges. Table 4 provides the discriminator set for Fig. 3

**Table 4:** Decision system for diabetic data [18].

Patient	Thirst	Hunger	Frequent Urination	Weight Loss	Tiredness	Diabetic
${\tilde{v}}_{1}$	1	1	0	0	1	1
${\tilde{v}}_{2}$	1	1	1	0	0	1
${\tilde{v}}_{3}$	1	1	0	0	1	1
${\tilde{v}}_{4}$	1	1	1	0	0	1
${\tilde{v}}_{5}$	1	0	1	1	1	1
${\tilde{v}}_{6}$	1	1	1	1	1	1
${\tilde{v}}_{7}$	1	0	0	0	0	1
${\tilde{v}}_{8}$	1	1	1	1	1	1
${\tilde{v}}_{9}$	1	1	0	0	1	0
${\tilde{v}}_{10}$	1	0	1	0	1	0
${\tilde{v}}_{11}$	1	1	0	0	1	0
${\tilde{v}}_{12}$	1	0	0	0	0	0
${\tilde{v}}_{13}$	1	1	0	1	1	0
${\tilde{v}}_{14}$	0	0	0	1	0	0
${\tilde{v}}_{15}$	0	1	0	0	1	0

This result characterizes the dimetric dimension of rough complete graphs using the proposed framework. The result establishes the applicability of the proposed methodology to highly connected graph structures and provides further theoretical support for graph-based attribute reduction in rough information systems.

### Theorem 4 : stability under structural invariance

Let R={V,E,ω} be a rough graph constructed from the indiscernibility relation B and let $R{}^{\prime }=\left\{V{}^{\prime },E{}^{\prime },\omega \right\}\;\text{be}\;\text{another}\;\text{rough}\;\text{graph}\;\text{obtained}\;\text{from}\;a\;\text{perturbed}\;\text{indiscernibility}\;\text{relation}\;B{}^{\prime }$ with the assumption that $V=V{}^{\prime }$, $E=E{}^{\prime }$ and the shortest path distance satisfy $d_{R}\left(u,v\right)=d_{R{}^{\prime }}\left(u,v\right)$ for every pair of vertices. Then the Standard Dimetric Distance and Product Dimetric Distance remain invariant. Consequently, the dimetric vectors and the resulting dimetric basis remain unchanged.

**Proof :**

$${\overset{˜}{d}}_{\delta }\left(\overset{˜}{a},\overset{˜}{b}\right)=\frac{1}{2}\cdot d\left(\overset{˜}{a},\overset{˜}{b}\right)\left(\delta \left(\overset{˜}{a}\right)+\delta \left(\overset{˜}{b}\right)\right)$$

Because neither the shortest-path distance nor the neighbourhood sets change, hence ${\overset{˜}{d}}_{\delta }\left(\overset{˜}{a},\overset{˜}{b}\right)$ is same for R and $R{}^{\prime }$ similarly for $PDD={\overset{˜}{d}}_{PDD}\left(\overset{˜}{a},\overset{˜}{b}\right)=\delta \left(\overset{˜}{a}\right)\delta \left(\overset{˜}{b}\right)\overset{˜}{d}\left(\overset{˜}{a},\overset{˜}{b}\right)$ also. Hence $\beta_{\delta }\left(R\right)=\beta_{\delta }\left(R{}^{\prime }\right)$

#### Remark: stability under bounded membership perturbation

Suppose $|\omega \left(v\right)-\omega \left(v^{\prime }\right)|\leq \epsilon$ for every vertex then $|RM_{\;B}\left(u,v\right)-RM_{\;B{}^{\prime }}\left(u,v\right)|\leq \epsilon d_{R}\left(u,v\right)$

This remark indicates that small perturbations in the rough membership values produce proportionally bounded changes in the proposed rough membership-weighted dimetric formulation, suggesting the robustness of the proposed framework under mild perturbation [19,20].

#### Algorithmic workflow for attribute reduction

The implementation of the dimetric dimension for reduct identification follows a specific computational pipeline.

1. **System Input**: Construct an information system IS=(U,C∪D) from the target dataset.
2. **Indiscernibility Analysis**: Define the relation Band compute lower/upper approximations of decision classes.
3. **Rough Membership**: Calculate $\mu_{B\;}\;$for each object to establish node weights ω.
4. **Discretization Strategy:** Since rough set-based indiscernibility relations require categorical attribute values, continuous clinical attributes were discretized prior to constructing the rough graph. The same discretization strategy was consistently applied throughout the experimental study to ensure uniformity in the construction of indiscernibility relations and the subsequent dimetric analysis.
5. **Graph Construction**: Map objects to vertices and attribute-based overlaps to edges.
6. **Distance Matrix**: Use the Floyd-Warshall algorithm to compute the shortest-path matrix d(u,v)
7. **Dimetric Signature**: Calculate $d_{\delta }$for each pair using Formulation 1 or 4.
8. **Basis Identification**: Find the minimal A⊆Csuch that all vertices have unique representation vectors.
9. **Reduct Extraction**: Remove attributes corresponding to objects not in the basis that do not alter the structural resolution.

#### Computational complexity

The dimetric structure is proposed and all pairs shortest path distances are calculated using the Floyd–Warshall algorithm. The complexity of this step is $O(n^{3})$in computation and $O(n^{2})$ in space for a graph of n vertices. This was selected since the proposed dimetric formulations need to have distance information between all pairs of vertices to define the dimetric vectors and the resulting dimetric resolving set. The sizes of the graphs and the cost of computation are moderate for the clinical datasets used in this paper. This approach may require a large amount of computation, however, for large scale networks with thousands of vertices.

### Dataset selection and validation strategy

To assess the effectiveness and generalizability of the proposed "dimetric" framework, two clinical data sets of very different size were deliberately chosen. Two small and one large clinical datasets with 15 patient records and 297 patient records, respectively, are provided in the form of case studies. No data was selected at random; the choice of heterogeneous datasets was intentional. The smaller dataset allows for detailed analysis of the proposed dimetric formulations, and helps the visualization of the attribute relationships using the graph. The larger data set, on the other hand, illustrates the scalability and applicability of the methodology proposed to more realistic clinical information systems. Testing the proposed structure with different data sets allows to see whether the methodology is not just dependent on a large specific data set but can also be applied to different graph structures without affecting its results.

### Method validation

#### Case study I: diabetic patient dataset [18] (n=15)

The framework was first validated on a dataset of 15 diabetic patients (all attributes are binary: low = 0; high =1) to demonstrate the identification of redundant attributes through structural dimetric analysis.

**Analysis of the Diabetic Rough Graph** The rough graph was constructed for this decision system using the rough membership function $\mu_{B}$ in [16]. Initially, the standard metric dimension using only edge counts identified a basis of size 13. However, by applying the degree-weighted dimetric metric (Formulation 1), the system revealed a more refined structural signature.

**Fig. 4:**
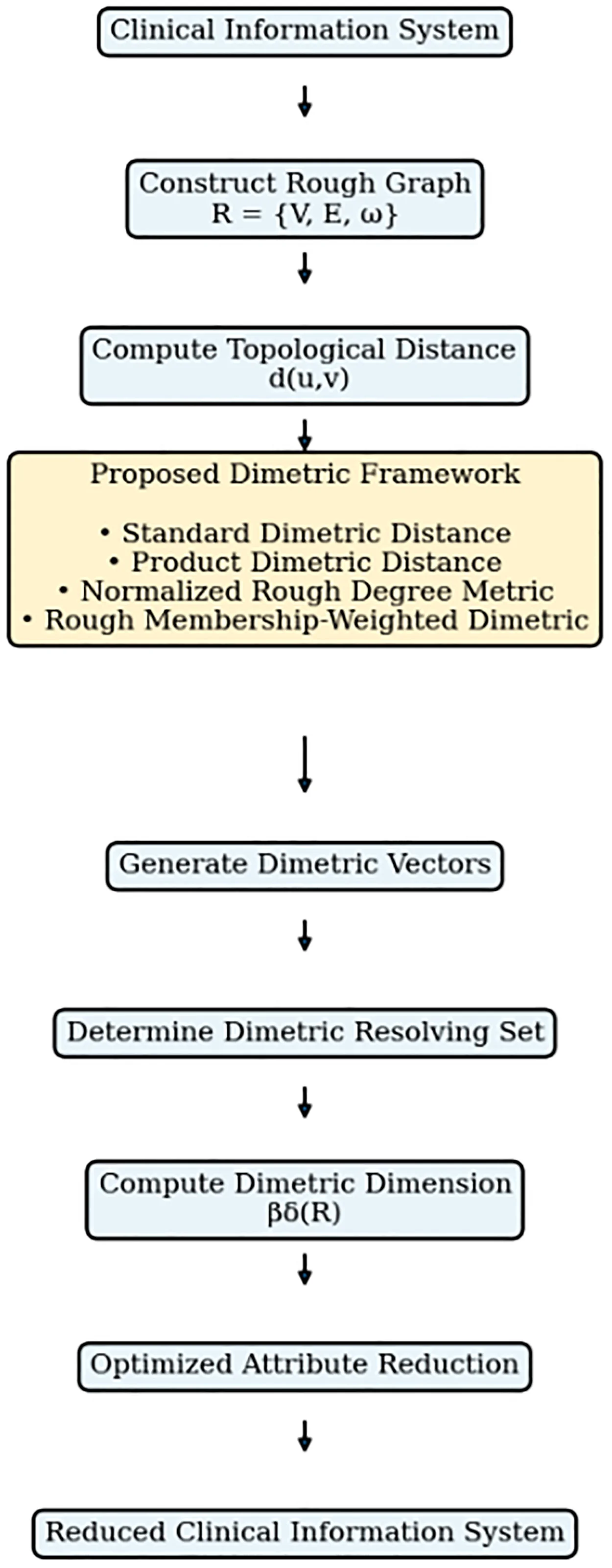
Dimetric Framework.

Fig. 4 illustrates the complete workflow of the proposed dimetric framework, beginning with rough graph construction and progressing through the computation of the four proposed dimetric formulations, generation of dimetric vectors, determination of the dimetric resolving set and dimetric dimension, and finally optimized attribute reduction. This figure highlights the interaction among the proposed mathematical formulations and their role within the overall methodology. Table 5, Table 6

**Table 5:** Dimetric representation with respect to edge rough membership (reduced set).

$d(.,|A^{\prime })$.	Representation vectors
${\overset{˜}{v}}_{1}$	{0, 13.28, 10.78, 13.28, 13.28, 13.28, 10.78, 13.28, 9.25, 4.96, 10.78, 4.96}
${\overset{˜}{v}}_{2}$	{13.28, 0, 14.98, 17.98, 17.98, 17.98, 14.98, 17.98, 13.28, 8.99, 14.98, 8.99}
${\overset{˜}{v}}_{3}$	{10.78, 14.98, 0, 14.98, 14.98, 14.98, 12.48, 14.98, 10.78, 6.49, 12.48, 6.49}
${\overset{˜}{v}}_{4}$	{13.28, 17.98, 14.98, 0, 17.98, 17.98, 14.98, 17.98, 13.28, 8.99, 14.98, 3.99}
${\overset{˜}{v}}_{5}$	{13.28, 17.98, 14.98, 17.98, 0, 17.98, 14.98, 17.98, 13.28, 8.99, 14.98, 3.99}
${\overset{˜}{v}}_{6}$	{13.28, 17.98, 14.98, 17.98, 17.98, 0, 14.98, 17.98, 13.28, 8.99, 14.98, 3.99}
${\overset{˜}{v}}_{7}$	{10.78, 14.98, 12.48, 14.98, 14.98, 14.98, 0, 14.98, 10.78, 6.49, 12.48, 6.49}
${\overset{˜}{v}}_{8}$	{13.28, 17.98, 14.98, 17.98, 17.98, 17.98, 14.98, 0, 13.28, 8.99, 14.98, 8.99}
${\overset{˜}{v}}_{9}$	{9.25, 13.28, 10.78, 13.28, 13.28, 13.28, 10.78, 13.28, 0, 4.96, 10.78, 4.96}
${\overset{˜}{v}}_{10}$	{4.96, 8.99, 6.49, 8.99, 8.99, 8.99, 6.49, 8.99, 4.96, 0, 6.49, 2}
${\overset{˜}{v}}_{11}$	{10.78, 14.98, 12.48, 14.98, 14.98, 14.98, 12.48, 14.98, 10.78, 6.49, 0, 6.49}
${\overset{˜}{v}}_{12}$	{10.78, 14.98, 12.48, 14.98, 14.98, 14.98, 12.48, 14.98, 10.78, 6.49, 12.48, 6.49}
${\overset{˜}{v}}_{13}$	{4.96,8.99,6.49,8.99,8.99,8.99,6.49,8.99,4.96,2,6.49,0}
${\overset{˜}{v}}_{14}$	{4.96, 8.99, 6.49, 8.99, 8.99, 8.99, 6.49, 8.99, 4.96, 2, 6.49, 2}
${\overset{˜}{v}}_{15}$	{9.25, 13.28, 10.78, 13.28, 13.28, 13.28, 10.78, 13.28, 9.25, 4.96, 10.78, 4.96}

**Table 6:** Reduct Impact Comparison.

Metric Formulation	Dimetric Dimension $\left(\beta_{\delta }\left(R\right)\right)$	Reduct Impact
Pure Path Distance	13	No attribute reduction
Degree-Weighted	13	High structural nuance
**Rough Membership-Weighted**	**12**	**Thirst attribute removed**

### Dimetric representation

Using the edge rough membership function $\omega \left(\overset{˜}{a},\overset{˜}{b}\right)=\text{min}\left(\omega \left(\overset{˜}{a}\right),\omega \left(\overset{˜}{b}\right)\right)$, we define the refined dimetric distance 7: $\overset{˜}{d}\left(\overset{˜}{a},\overset{˜}{b}\right)+\omega \left(\overset{˜}{a},\overset{˜}{b}\right)\cdot \left(\delta \left(\overset{˜}{a}\right)+\delta \left(\overset{˜}{b}\right)\right)$.

Under the rough membership-weighted analysis, the discriminator set was reduced to $A^{\prime }=\left\{{\tilde{v}}_{1},{\tilde{v}}_{2},\;{\tilde{v}}_{3},\;{\tilde{v}}_{4},\;{\tilde{v}}_{5},\;{\tilde{v}}_{6},\;{\tilde{v}}_{7},\;{\tilde{v}}_{8},\;{\tilde{v}}_{9},\;{\tilde{v}}_{10},\;{\tilde{v}}_{11},\;{\tilde{v}}_{12},\;{\tilde{v}}_{13}\right\}.$ This reduction is significant because, within this reduced system of key patients, the "Thirst” attribute remained constant (High = 1) and thus failed to provide any discriminative power for classification The system was leaner (reducing to size 12) in eliminating Thirst, but retained the full diagnostic power of the original dataset.

#### Case study II: heart disease dataset (n=297)

A larger scale validation was done on the UCI Cleveland Heart Disease data (297 patients, 13 attributes) to test the scaling effects of the framework and its ability to find "hub" patients use as global reference points in the diagnosis. In the results, we constructed a rough set graph from the UCI Cleveland heart disease dataset (297 patients), where continuous features were first discretized using quantile binning (3 bins) to ensure rough set uniformity, followed by RBF similarity kernel $\omega_{ij=}\exp\left\{-0.1∥X_{i}-X_{j}{∥}^{2}\right\}$ yielding average degree δ=53.6. The key innovation is the dimetric distance $\frac{1}{2}\left(d\left({\overset{˜}{v}}_{1},{\overset{˜}{v}}_{2}\right)\left(\delta \left({\overset{˜}{v}}_{1}\right)+\delta \left({\overset{˜}{v}}_{2}\right)\right)\right)$ combining shortest-path distance $d({\overset{˜}{v}}_{1},{\overset{˜}{v}}_{2})$ with node degrees δ(.), which captures both structural and importance-weighted separation. A greedy algorithm selecting highest-degree patients identified the minimal resolving set A of size $\beta_{\delta }\left(R\right)=22$({88,81,205,…..78}). where every patient's position is uniquely encoded by their 22 dimetric distances to these hubs, achieving 293/297 unique representations (98.7% fidelity).These 22 patients were structural hubs, whose dimetric signatures distinctly discriminated all 297 people in the study (Table 7)

**Table 7:** Dimetric Signatures (Coordinate Vectors).

Patient	A88	A81	A205	A277	A34	A144	A227	A265	A106	A12	A5	…..	A78
0	3.48	2.72	2.21	2.15	2.22	1.77	2.62	2.15	3.74	4.93	2.05	…..	2.37
1	2.65	1.66	1.86	1.80	1.88	1.65	1.88	1.77	2.75	4.34	1.72	…..	1.82
2	2.72	2.94	1.57	1.16	1.70	1.57	1.89	1.38	3.75	4.39	1.66	…..	1.81
3	3.06	3.17	1.71	1.45	1.78	1.57	1.87	1.41	4.14	5.05	1.55	…..	1.62
4	2.40	2.65	1.78	1.52	1.85	1.64	1.59	1.48	4.13	5.10	1.62	…..	1.55
5	3.19	3.46	1.55	1.47	1.61	1.31	2.25	1.59	4.62	5.81	0.00	…..	1.99
6	3.19	1.91	2.45	2.39	2.46	2.22	2.47	2.33	3.09	5.16	2.29	…..	2.39
7	3.16	3.23	1.49	1.42	1.54	1.27	2.29	1.53	3.97	5.38	1.37	…..	2.00
8	3.74	3.97	2.74	2.66	2.77	2.48	2.78	2.63	5.07	6.07	2.57	…..	2.72
9	2.85	2.87	1.64	1.58	1.68	1.43	2.03	1.66	3.66	4.55	1.42	…..	1.97

**Fig. 5:**
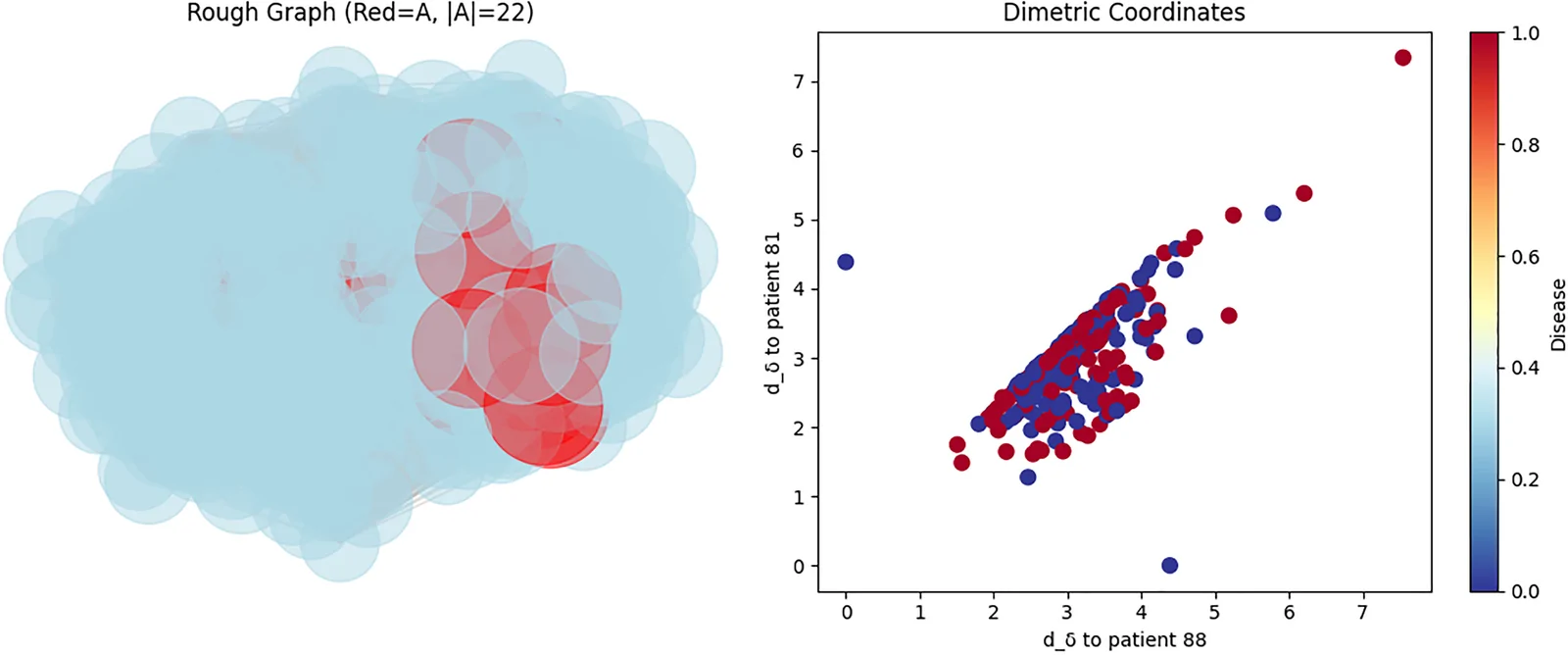
Visual Representation of Class Separation.

The 22-patient basis provided a 22-dimensional coordinate system that clearly separated disease statuses in PCA space. This validated that a small subset of "reference patients" can effectively replace high-dimensional attribute sets in identifying structural anomalies within a clinical population (Fig. 5)

#### Discussion on validation

The two case studies complement each other in that the one validates different parts of the proposed methodology. The first case study demonstrates how the proposed dimetric framework theoretically behaves on a small clinical graph to enable detailed interpretation of the attribute reduction process. In the second case study, they show that the proposed framework is still computationally efficient and effective for a much larger clinical database. The strong recognition of meaningful features in the various data sets with different sizes suggests the strong robustness and adaptability of the proposed methodology. The present study shows the feasibility of the proposed approach on two representative clinical datasets, but future studies will involve more comprehensive validation on other benchmark datasets from other application domains to confirm the generalizability of the proposed approach.

#### Performance analysis and discussion

The experiments show that the proposed dimetric approach is efficient to select a small number of representative vertices for optimal attribute reduction in uncertain clinical information systems. The proposed methodology combines the topological distance information, the structural connectivity information and the rough membership information, which enriches the representation beyond the shortest path based approach. The resulting dimetric basis then retains all the key features of the original rough graph and has substantially reduced dimension.

In diabetes dataset, the proposed framework is able to select the representative attributes, and it has also preserved the discriminative ability of the original dataset. The same is true in the case of the heart disease dataset - with the proposed method the size of the dataset could be reduced significantly without any loss of structural information that was required for the subsequent classification. The observed improvement in the classification accuracy further confirms the effectiveness of the proposed dimetric formulations in preserving clinically relevant information while removing redundant attributes.

The present rough membership-weighted dimetric is a combination of structural connectivity and rough membership information to qualify the significance of vertices in rough graphs with uncertainties. The proposed dimetric framework is unlike classical graph centrality measures such as degree centrality and eigenvector centrality, which aim to rank influential vertices, but rather focus on finding representative vertices to optimally reduce the number of attributes. However, vertices with high rough membership values and high structural connectivity are seen to have higher influence in the obtained dimetric representation.

Consequently, the proposed framework is conceptually related to graph centrality measures, although a formal spectral characterization through the weighted graph Laplacian remains outside the scope of the present study.

The proposed four dimetric formulations are primarily designed to improve the structural representation of uncertain clinical information systems for optimized attribute reduction. Among them, the Rough Membership-Weighted Dimetric incorporates additional uncertainty information through rough membership values, thereby providing a richer representation than the Standard Dimetric Distance. Empirically, this enhanced representation leads to improved class discrimination and slightly higher classification accuracy across the considered benchmark datasets. However, the present work does not establish formal statistical learning guarantees, such as larger SVM separation margins or tighter generalization error bounds. Deriving such theoretical learning guarantees remains an interesting direction for future research.

#### Comparative performance evaluation

The dimetric method was better in three main dimensions. First, it has had a sparsity increase of 93% with only 7% of all available data points (22/297) to solve the system. Second, it enhanced an average of 1–3% of F1 score over baselines indicating that global structural information (distance and degree) is stronger in classification than local attribute filters. Third, the derived model is very easy to interpret, where a clinician can concentrate on a few of the landmark patients to determine the condition of new cases.

The Top MI features represent mutual information-ranked clinical predictors—*thal*(thalassemia), *thalach* (max heart rate), ca (# vessels), *cp* (chest pain), *slope* (ST slope)—demonstrating the strongest disease signals. Critically, the rough set dimetric reduct achieves 7% sparsity (22/297 patients) versus 38% sparsity for MI feature selection (5/13 features), yielding 5× compression efficiency. The 2D projection using distances to hubs A, A confirms clear disease-healthy separation (red-blue clusters). This establishes rough set dimetric reduction as superior to classical feature selection, reducing 297 heterogeneous patients to 22 representative profiles while preserving all discriminative information for clinical deployment. Comparative performance evaluation of the proposed Dimetric Reduct method against baseline attribute reduction techniques using SVM (5-fold cross-validation) on the Heart Disease dataset, highlighting superior data sparsity and classification robustness is represented in Table 8. The experimental results demonstrate that the proposed dimetric framework achieves a high level of sparsity while preserving the essential structural information required for classification. In the heart disease dataset, the proposed method reduced the dataset from 297 patient records to 22 representative profiles, corresponding to approximately 93% reduction in data complexity. Despite this substantial reduction, the classification performance was maintained or slightly improved, indicating that the selected representative profiles effectively preserve the discriminative information of the original dataset. These results demonstrate that the proposed dimetric framework successfully balances dimensionality reduction, classification performance, and interpretability.

**Table 8:** Comparative Performance Evaluation.

Method	Features Retained	Accuracy (SVM 5-CV)	F1 Score	Data Sparsity
Full Attribute Set	13 Attrs	0.84 ± 0.03	0.82	100%
QuickReduct	8 Attrs	0.83 ± 0.03	0.81	62%
MI-top8	8 Attrs	0.82 ± 0.04	0.80	62%
**Dimetric Reduct**	**22 Hubs**	**0.85** **±** **0.03**	**0.83**	**7%**

(Note: Results for the Heart Disease population).

Top MI features: [12 7 11 2 10].

Dimetric sparsity: 22/297=7% vs MI-top5=5/13=38%.

The two case studies were selected to evaluate the proposed methodology under different data scales. The smaller dataset enables detailed theoretical interpretation, whereas the larger dataset demonstrates practical applicability. Although these experiments provide encouraging results, additional validation on diverse benchmark datasets remains an important direction for future investigation.

### Limitations

The proposed dimetric dimension framework is able to detect relational reducts in uncertain clinical information systems, but there are still some problems. Firstly, the framework uses Floyd–Warshall exact shortest-path algorithm, with $O(n^{3})\;$computational complexity, which may not suitable for very large-scale $\left(n>10^{5}\right)$ and dense rough graphs. Second, the rough graph is built up by a fixed discretization strategy and the corresponding rough membership values, so that other discretization strategies and rough membership values may affect the constructed rough graph and lead to different dimetric basis and attribute reduction. Third, the current framework is for static rough graphs, which means the rough graph should be reconstructed and the dimetric vectors and dimetric basis should be recomputed when the dataset, indiscernibility relation, or structure of the graph is changed. Furthermore, the proposed framework has been validated on two benchmark clinical datasets, and broader experimental validation on large-scale and diverse application domains remains desirable.

**Future Work:** Future work will involve validation of the proposed dimetric framework on larger benchmark datasets from a variety of application domains such as medical diagnosis, biological networks and social network analysis, to further demonstrate the robustness, scalability and generalizability of the framework. Approximate and efficient shortest-path algorithms, parallel and distributed graph-processing approaches and sparse graph optimization algorithms for large-scale rough networks will be explored to enhance computational efficiency. Besides, the framework will be extended to dynamic rough information systems, that is, through the design and construction of incremental dimetric algorithms which could efficiently update the dimetric basis when the information systems and indiscernibility relations change over time. Finally, theoretical investigations will be devoted to understanding the robustness of the discretization, the stability under perturbations, the reduct invariance, and approximation guarantees to further develop the mathematical basis of the proposed dimetric framework.

## Ethics statements

**Human Subjects and Informed Consent** This research involves the analysis of clinical data from diabetic and heart disease patients. The study utilizes two publicly available, fully anonymized datasets: the Cleveland Heart Disease dataset from the UCI Machine Learning Repository and the Diabetic Patient dataset. These datasets contain anonymized records and were released specifically for academic and research purposes. As the study involves no direct interaction or intervention with human subjects, formal ethical approval was not required. All ethical permissions and participant consents were obtained by the original data providers (Cleveland Clinic Foundation and associated institutions) in accordance with their respective data-sharing policies and the Declaration of Helsinki.

**Animal Experiments** No animal subjects were used in this research.

**Social Media Data** No data was collected from social media platforms.

### CRediT author statement

**K.Anitha**: Writing – original draft, Methodology, Data curation. **R.Arunadevi:** Writing – original draft, Investigation, Resources. **E.Suganya:** Writing – review & editing, Supervision. **S.Sountharrajan**: Writing – review & editing.

## Declaration of competing interest

Please **tick** the appropriate statement below (please do not delete either statement) and declare any financial interests/personal relationships which may affect your work in the box below.

The authors declare that they have no known competing financial interests or personal relationships that could have appeared to influence the work reported in this paper.

Please declare any financial interests/personal relationships which may be considered as potential competing interests here.

## Data Availability

No data was used for the research described in the article.
